# Functional outcome of tarsometatarsal joint fracture dislocation managed according to Myerson classification

**Published:** 2014

**Authors:** Xiao YU, Qing-Jiang PANG, Chang-Chun YANG

**Affiliations:** 1 Dr. Xiao YU, PhD, Department of Orthopedics, Ningbo No.2 Hospital, Ningbo, 315010, Zhejiang, China.; 2 Dr. Qing-Jiang PANG, PhD, Department of Orthopedics, Ningbo No.2 Hospital, Ningbo, 315010, Zhejiang, China.; 3 Dr. Chang-Chun YANG, MD, Department of Orthopedics, Ningbo No.2 Hospital, Ningbo, 315010, Zhejiang, China.

**Keywords:** Tarsometatarsal joint, Fracture-dislocation, Open reduction, Internal fixation

## Abstract

***Objective*** ***:*** To summarize the functional outcome of tarsometatarsal joint fracture-dislocation managed according to Myerson classification.

***Methods*** ***:*** Total eighty cases of tarsometatarsal joint fracture-dislocation were treated from Mar 2004 to Feb 2012. According to the Myerson classification, there were 14 cases in type A, 12 cases in type B1, 28 cases in type B2, 11 cases in type C1 and 15 cases in type C2. All the cases were treated with open reduction and internal fixation and the incisions and implants were also selected according to the Myerson classification. X-ray was examined during the follow-up period and functional evaluation was carried out by American Orthopaedic Foot and Ankle Society (AOFAS) midfoot score system. Analysis of variance was used to test the different types of Myerson classification.

***Results*** ***:*** Sixty eight patients got a mean follow-up of 24 months (15-36 months). No patient suffered from infection, skin flap necrosis and X-ray showed there were no implants loosening or breakage. The mean AOFAS score was 88.4(47-100) and excellent and good result was 89.7%. The differences among Myerson classifications showed that there were statistical significance between type B and type A, type C (*P*<0.05) Three patients suffered from severe pain and difficult walking, X-ray showed the ambiguity of the joint space, which can be diagnosed as posttraumatic arthritis. One patient had arthrodesis finally.

***Conclusion*** ***:*** The Myerson classification is helpful to make preoperative plan and judging prognosis to the tarsometatarsal joint injuries. In type B, single or double incisions with screw or plate fixation is enough, while in type A and type C, double or triple incisions with screw or plate fixation in medial joints and Kirschner wire fixation in lateral joints are needed. Postoperatively, the type B patients had better prognosis than type A and type C patients. However, the concomitant injuries around the tarsometatarsal joint were not included in Myerson classification, which is the limitation but cannot be neglected.

## INTRODUCTION

Tarsometatarsal joint fracture-dislocation (also known as Lisfranc injury) is always caused by traffic accidents, fall from height and other high-energy injuries.^1^ Though it is seldom seen in clinical (accounting for less than 1% of all orthopedic trauma), missed diagnosis and diagnostic errors frequently occur since the anatomical structures in the damaged positions and the mechanical transduction process are complex, and high-energy injuries are always concomitant with injuries in other parts of the foot.^2^ It was reported that the missed diagnosis on initial presentation was in approximately 20% of cases.^3^ Once the missed Lisfranc injuries cause malunion and traumatic arthritis of the tarsometatarsal joint, it will affect the stress transduction in the foot and result in abnormal gait with symptoms of pain and permanent disability.^4^^,^^5^ Therefore, therapeutic requirements and difficulties are high.

At present, the Myerson classification is widely used in Lisfranc injuries, which clarified the injury mechanism.^6^ However, seldom literature linked the Myerson classification with preoperative plan and prognosis. In this study, we retrospectively analyzed 80 patients functional outcomes of tarsometatarsal joint fracture-dislocation in order to illustrate the strategies for surgical treatments of these injuries according to Myerson classification.

## METHODS

***General information: ***A total of 80 pataients (52 males and 28 females) of tarsometatarsal joint fracture-dislocation were treated from March 2004 to February 2012. The age ranged from 25 years to 64 years (43.2 years old in average). 68 patients had closed injuries and 12 patients were open injuries. Cause in 33 patients was traffic accidents, 24 patients had fall from height, 13 patients had crash with heavy things and 10 cases were crushed on machines. All these patients were subjected to X-ray, CT scan and three-dimensional reconstruction and uninjured side control was also set up. According to the Myerson classification^6^, there were 14 cases in type A, 12 cases in type Bl, 28 cases in type B2, 11 cases in type Cl and 15 cases in type C2. In addition seven patients had concomitant fractures in metatarsals, 12 patients had unstable intercuneiform articulations, and 5 patients with unstable cuneonavicular joint. All the patients were subjected to open reduction and internal fixation (ORIF).The time duration between injury and surgery ranged from 1.5 days to 10 days (5.5 days in average), however, the open injuries were treated in emergency by debridement and Kirschner wire for temporary fixation prior to the ORIF.

**Fig.1:**
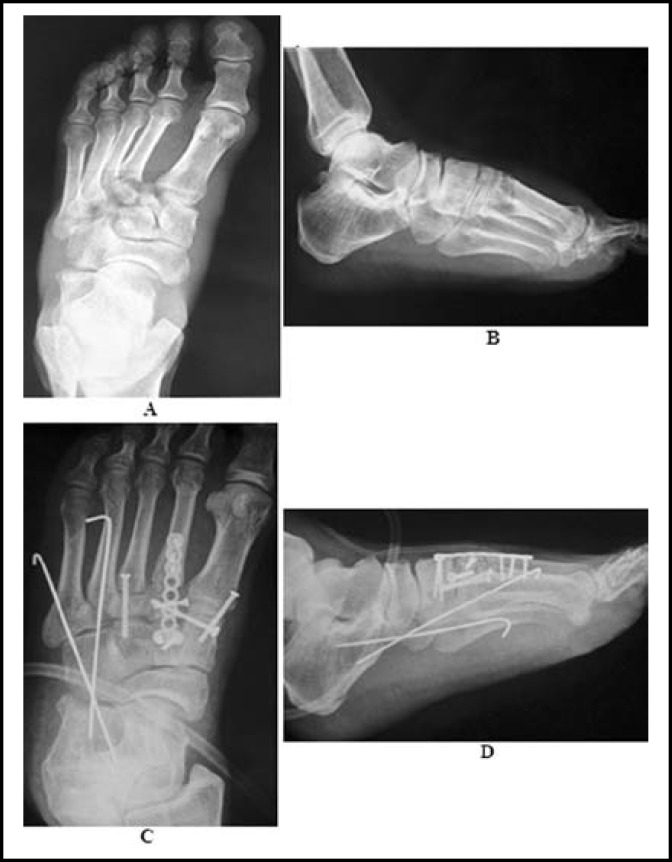
A male patient with Left foot total tarsometatarsal joint fracture dislocation caused by a traffic accident. **(A, B)** The X-ray showed a Myerson type A injury. **(C, D)** The postoperative X-ray showed a good reduction and fixation

**Fig.2:**
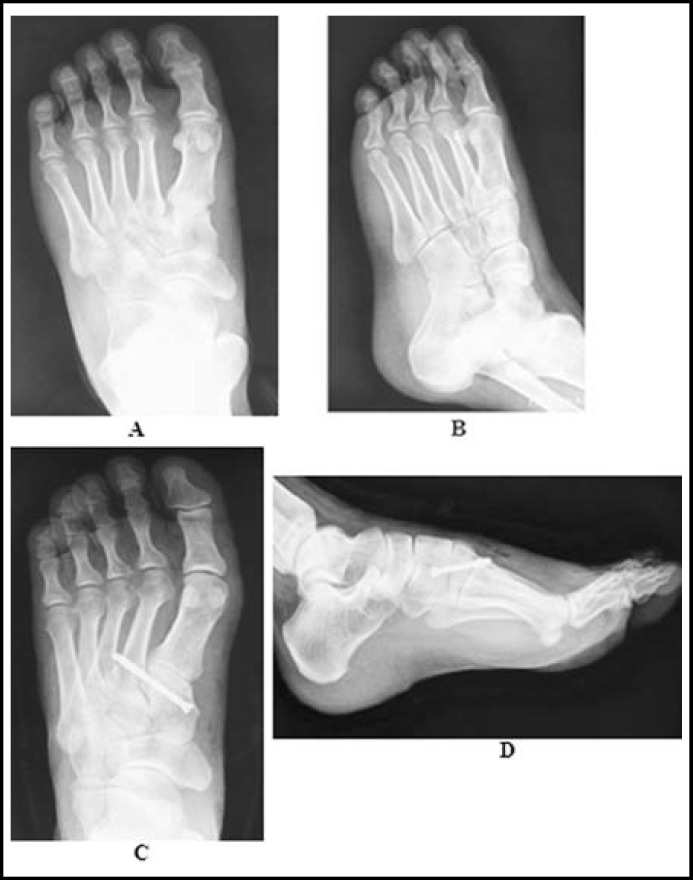
A female patient with second tarsometatarsal joint fracture dislocation in left foot caused by fall from height. **(A, B)** The X-ray showed a Myerson type B2 injury with the widen space between the base of first and second metatarsals. **(C, D)** The postoperative X-ray showed a good reduction and fixation

***Surgical treatment: ***The operations were carried out after intraspinal anesthesia. Incision was determined according to Myerson classification. To Myerson type A patients, double or triple incisions were selected. Additional attention was paid to check whether vassels or nerves were compacted at the fractures or articular facet before reduction to prevent the iatrogenic injuries.^7^ The second tarsometatarsal joint should be firstly reduced, if the fracture is relatively intact, 3.5 mm-cortical bone screws or 4.0 mm-cannulated screws were used to fix the intermediate cuneiform bone from the base of the second metatarsals via the second tarsometatarsal joint. Afterwards, a “Lisfranc screw” was placed from the medial side of medial cuneiform towards the base of the second metatarsal along with the Lisfranc ligament.^8^ If the space between medial cuneiform bone and intermediate cuneiform bone was widened and the screws are difficult for fixation, dorsal plates can be selected for fixation.^9^ Normally, once the first and second tarsometatarsal joints were reduced, reduction of the remaining tarsometatarsal joints would be easier. The base of the third metatarsal can be fixated to the intermediate or lateral cuneiform. Finally, Kirschner wire was used to successively fixthe base of the fourth and the fifth metatarsals to the lateral cuneiform and the cuboid.^10^ In Myerson type B1 patients, the incision was always produced by the dorsal of the first tarsometatarsal joint, dorsal plate was used for fixation. To Myerson type B2 patients, the first incision can be between the second and the third metatarsals to facilitate the probing of intermediate column, while the therapeutic program for Myerson type C1 or C2 patients was almost similar to that for type A patients. To the patients concomitant with injuries around the tarsometatarsal joint, the original incision can be extended on the basis to expose the injured position, and reduction fixation was then carried out. (The typical cases are shown in Fig. 1 and 2).

***Postoperative management: ***The limbs were lifted and adjunctive therapy with antibiotics, antioncotics and dressing changing was carried out to prevent the infections, swelling and skin flap necrosis. The patients were treated by a non weight-bearing cast for 6 weeks and a partial weight-bearing cast for another 6 weeks supplemented with rehabilitation exercises.^11^ Regular follow-up was asked and the functional recovery was evaluated according to the American Orthopaedic Foot and Ankle Society (AOFAS) midfoot score system. Postoperatively, X-ray examination was carried out once a month during the first three months then it could be taken at the sixth month and the twelfth month to evaluate the efficacy of reduction maintenance and judge whether the implants can be taken out.^12^ Kirschner wires and screws were removed within three months postoperatively, afterwards weight-bearing walk was gradually allowed. The plates were always removed one year postoperatively.

***Statistical method: ***The results of AOFAS scoring were described by ±S. Variance analysis was carried out for the comparison in the interclass difference, and *P*＜0.05 indicated that the difference was statistically significant.

## RESULTS

Sixty eight patients accepted follow-up for 15-36 months (24 months in average) and the postoperative complications such as infection, skin flap necrosis, loosening or breakage of the implants, malunion and metastatic metatarsalgia were not detected.

At the time of one-year postoperative follow-up, all the 68 patients were evaluated by the AOFAS scoring system. The scores ranged from 47 to 100 in the 68 patients and the average score was 88.4. According to the AOFAS score, the result was excellent in 35 cases, good in 26 cases, 4 cases in fair and poor in three cases. The combined excellent and good result was seen in 89.7%. The AOFAS score was 85.3 in Myerson type A patients, 93.2 in type B patients and 82.8 in type C patients respectively. The excellent and good result in type A and type C were 80% and 79.2%, while in type B it was as high as 100%. The variance analysis showed that the differences in the scores of Myerson type B were statistically significant in comparison to those of Myerson type A and type C (*P*<0.05), however, no statistical significance was found between Myerson type A and type C(*P*>0.05). (Table-I)

Three patients (one in type A and two in type C) got poor evaluation and suffered from obvious pain and dysfunction, the X-ray showed that the joint space was blurred and they were diagnosed as traumatic arthritis, two of which got pain relief after treatment and the other patient accepted tarsometatarsal joint arthrodesis.

**Table-I:** The functional outcomes of different Myerson types according to the AOFAS score

***Myerson Types***	***AOFAS Score***	***Excellent***	***Good***	***Fair***	***Poor***
Type A	85.3±8.7	6	2	1	1
Type B	93.2±4.8*	23	11	0	0
Type C	82.8±11.4^▲^	6	13	3	2
Total	88.4±8.9	35	26	4	3

* Compared with Myerson type A, *P*<0.05,

▲ Compared with Myerson type B, *P*<0.05

## DISCUSSION

Tarsometatarsal joint connects the forefoot and midfoot, whose injuries may seriously affect configuration and mechanical transduction of feet, therefore, anatomical reduction is required to recover a painless and stable plantigrade foot.^13^ Since articular facet disintegration, and soft tissue compaction are always detected due to high-energy injuries, it is difficult for closed reduction, moreover, some researchers have pointed out that excellent and good could be 50%-95% VS. 17%-30% whether the anatomical reduction could be achieved or not.^10^ Thus, almost all patients require open reduction.

The surgical target is different because of different structure and function in “three columns” theory in tarsometatarsal joint. From the anatomical and functional view, the medial and intermediate columns play predominant roles in maintaining the inelasticity of foot and absorbing shock compared with the lateral column in balancing the weight-bearing on forefoot. Therefore, we used screw or plate fixation in the medial and intermediolateral columns to reduce the effects of increased activities on mechanical transduction in midfoot. While in the lateral column, Kirschner wire was used to avoid joint stiffness postoperatively.^14^ Attention should be paid that the screw provide trans-articular facet fixation, which may damage articular cartilages and secondary to the traumatic arthritis. Moreover, it is always difficult in patients with comminuted articular facet. These defects can be overcome by plate, which is suitable for the cases with comminuted articular facet.^9^

In this study, longitudinal incisions were used. In Myerson type A and type C patients, we always selected double or triple incisions. In double-incision cases, the first incision was between the first and the second metatarsals, and the second incision was between the fourth and the fifth metatarsals. If triple incisions were selected, the first incision was at the second tarsometatarsal joint and the second incision was between the third and the fourth metatarsals, while the third incision could be between the fourth and the fifth metatarsals. In type B1 patients, since only the medial column was involved, we selected the incision at the dorsal or medial side of the first tarsometatarsal joint without affecting neurovascular bundles.^7^ In type B2 patients, it also required double incisions, since the medial columns were not injured, the first incision can be selected between the second and the third metatarsals. Compared with the incisions introduced by Zgonis,^15^ we adjusted the medial incision between the first and the second metatarsals to facilitate removal of any interposed soft tissues from the articulation.

Except the selection of incision, we also found Myerson classification had indicative function for judging prognosis. In this study, the AOFAS scores in type A, type B type, C patients were 85.3, 93.2 and 82.8 respectively. The variance analysis showed the differences among type B, type A and type C were statistically significant. However, there were no statistical significance between type A and type C. The reason may be that type A and type C patients were mostly injured in three columns, particularly in type A injuries, though dislocation towards one direction and it was relatively easier for reduction and fixation, the initial injuries were more severe than type B and even accurate anatomical reduction may lead to certain influences on the prognosis. Better prognosis means less postoperative complications. Postoperative complications of tarsometatarsal joints injuries mainly include malunion, metastatic metatarsalgia, joint degeneration and traumatic arthritis.^16^ The malunion and metastatic metatarsalgia is mostly because of the early weight-bearing that lead to the abnormal stress transduction in the foot. In this study, we treated the patients postoperatively with non-weight bearing cast and partial weight bearing cast for three months. In some type A or type C patients with comminuted fractures, the non-weight bearing time may be even longer, therefore, no patient suffered from malunion and metastatic metatarsalgia. Joint degeneration and traumatic arthritis are the most frequently seen complications. In this study, we treated 28 patients with comminuted fractures and three patients (one in type A and two in type C) suffered from traumatic arthritis at during follow-up. The symptoms in two patients were significantly improved after treatments; only one patient was subjected to secondary tarsometatarsal joint arthrodesis and postoperative intractable pain was also significantly improved. As regards comminuted injuries, researchers are apt to the arthrodesis. Reinhardt et al.^17^ treated 25 patients of Lisfranc injuries with arthrodesis and reported an average of 81 points of the AOFAS score and 84% satisfaction rate. However, three patients suffered from arthritis of adjacent joints. Sheibani-Rad et al.^18^ performed a systematic review to compare the arthrodesis and ORIF for Lisfranc injuries. They reported the AOFAS score in arthrodesis was 88 and the ORIF was 72.5. In our study, the mean AOFAS score was 88.4, which reflected good functional outcome with ORIF according to Myerson classification.

The Myerson classification put the emphasis on the injuries to the tarsometatarsal joint, however, it has the limitations because the injuries around the joint such as metatarsals and cuneonavicular joint were not included. In fact, tarsometatarsal joint complex is composed of tarsometatarsal joints, cuneonavicular joint, intercuneiform articulations and other joints. They have the functional cooperativity and should be taken as an entity. Therefore, these injuries should be considered when making the preoperative plan including selecting the extended incisions and longer implants. ^19^ In this study, 7 cases were concomitant with fractures in head or diaphysis of metatarsals, 12 patients were concomitant with unstable intercuneiform articulations, and 5 patients were concomitant with unstable cuneonavicular joint. The initial injuries of the 24 patients were relatively serious. We also carried out fixation for the injuries as mentioned above respectively during the fixation for tarsometatarsal joints, after follow-up, traumatic arthritis in tarsometatarsal joints was detected in three patients, but cuneonavicular joint or intercuneiform articulations was not obviously involved. This shows that timely surgical intervention may still improve the the prognosis in these patients with serious initial injuries.

## CONCLUSION

Tarsometatarsal joint fracture-dislocation is an easily overlooked injury, which will cause abnormal transduction of the stress from midfoot to forefoot. Therefore, the surgical treatment is essential to obtain anatomical reduction. In this study, we used ORIF according to the Myerson classification. In Myerson type B patients, single or double incisions with screw or plate fixation is enough, while to type A and type C, double or triple incisions with screw or plate fixation in medial joints and Kirschner wire fixation in lateral joints are needed. Postoperatively, the type B patients also had better prognosis and less complications than type A and type C patients. However, the concomitant injuries around the tarsometatarsal joint were not included in Myerson classification, which is the limitation of our study.

## Authors Contributions:

**X Y** designed the subject and did the manuscript writing **Q-J P** did the operation and edited the final manuscript  **C-C Y** followed up the patients and collected the X-ray films.
